# Supplementary material for: FOXL2 is a Progesterone Target Gene in the Endometrium of Ruminants
Source: Int J Mol Sci. 2020 Feb 21;21(4):1478. doi: 10.3390/ijms21041478 (PMC7073057; doi:10.3390/ijms21041478)
Supplement: Supplementary file 1 [file ijms-21-01478-s001.pdf]

International Journal of
Molecular Sciences

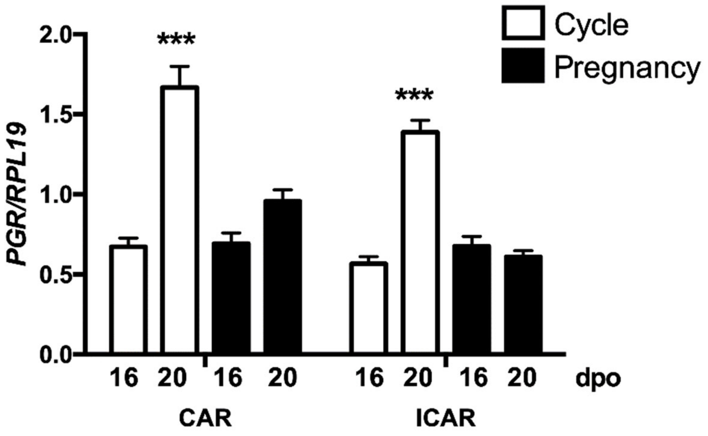

Figure S1. PGR transcript expression in cyclic and pregnant bovine endometrium. CAR and ICAR endometrial areas were collected from cyclic (n = 5 at Day 16; n = 6 at Day 20) and pregnant (preg; n = 4 at Day 16; n = 5 at Day 20) cross-bred beef heifers as previously published in [1]. Quantification of *PGR* mRNA by RTqPCR. Expression of *PGR* was normalized to that of RPL19. Quantitative data are means +/- s.e.m. ***: $p < 0.001$.

Supplementary Material and methods:

PGR expression during the period of pregnancy recognition. Cyclic and pregnant cross-bred beef heifers were synchronized and artificially inseminated as previously described [2]. The day of estrus was considered Day 0. Heifers were slaughtered at Day 16 (cyclic: n.5; pregnant: n.4) and at Day 20 (cyclic: n.6; pregnant: n.5). Uteri were collected, flushed, and, when present, recovered concepti were observed by microscopy to confirm the stage of development [3]. From pregnant and cyclic animals, endometrial CAR and ICAR areas were dissected from the uterine horns ipsilateral to the corpus luteum as previously described [4]. mRNA was extracted and cDNA quantification was performed as previousy published [1]

Supplementary references

1. Eozenou, C.; Vitorino Carvalho, A.; Forde, N.; Giraud-Delville, C.; Gall, L.; Lonergan, P.; Auguste, A.; Charpigny, G.; Richard, C.; Pannetier, M.; et al. FOXL2 Is Regulated During the Bovine Estrous Cycle and Its Expression in the Endometrium Is Independent of Conceptus-Derived Interferon Tau. *Biol. Reprod.* **2012**.
2. Forde, N.; Carter, F.; Spencer, T. E.; Bazer, F. W.; Sandra, O.; Mansouri-Attia, N.; Okumu, L. A.; McGettigan, P. A.; Mehta, J. P.; McBride, R.; et al. Conceptus-induced changes in the endometrial transcriptome: how soon does the cow know she is pregnant? *Biol. Reprod.* **2011**, *85*, 144–156.
3. Degrelle, S. A.; Campion, E.; Cabau, C.; Piumi, F.; Reinaud, P.; Richard, C.; Renard, J. P.; Hue, I. Molecular evidence for a critical period in mural trophoblast development in bovine blastocysts. *Dev. Biol.* **2005**, *288*, 448–460.
4. Mansouri-Attia, N.; Aubert, J.; Reinaud, P.; Giraud-Delville, C.; Taghouti, G.; Galio, L.; Everts, R. E.; Degrelle, S.; Richard, C.; Hue, I.; et al. Gene expression profiles of bovine caruncular and intercaruncular endometrium at implantation. *Physiol. Genom.* **2009**, *39*, 14–27.
